# Brain Energy Metabolism in Two States of Mind Measured by Phosphorous Magnetic Resonance Spectroscopy

**DOI:** 10.3389/fnhum.2021.686433

**Published:** 2021-06-28

**Authors:** Malik Galijašević, Ruth Steiger, Milovan Regodić, Michaela Waibel, Patrick Julian David Sommer, Astrid Ellen Grams, Nicolas Singewald, Elke Ruth Gizewski

**Affiliations:** ^1^Department of Neuroradiology, Medical University of Innsbruck, Innsbruck, Austria; ^2^Neuroimaging Research Core Facility, Medical University of Innsbruck, Innsbruck, Austria; ^3^VASCAge-Research Centre on Vascular Ageing and Stroke, Innsbruck, Austria; ^4^Department of Otorhinolaryngology, Medical University of Innsbruck, Innsbruck, Austria; ^5^Department of Radiation Oncology, Medical University of Vienna, Vienna, Austria; ^6^Yogamood, Innsbruck, Austria; ^7^Department of Pharmacology and Toxicology, Institute of Pharmacy and CMBI, Leopold Franzens University, Innsbruck, Austria

**Keywords:** meditation, brain energy metabolism, magnetic resonance, spectroscopy, phosphorous

## Abstract

**Introduction:** Various functional neuroimaging studies help to better understand the changes in brain activity during meditation. The purpose of this study was to investigate how brain energy metabolism changes during focused attention meditation (FAM) state, measured by phosphorous magnetic resonance spectroscopy (^31^P-MRS).

**Methods:** ^31^P-MRS imaging was carried out in 27 participants after 7 weeks of FAM training. Metabolite ratios and the absolute values of metabolites were assessed after meditation training in two MRI measurements, by comparing effects in a FAM state with those in a distinct focused attention awake state during a backwards counting task.

**Results:** The results showed decreased phosphocreatine/ATP (PCr/ATP), PCr/ inorganic phosphate (Pi), and intracellular pH values in the entire brain, but especially in basal ganglia, frontal lobes, and occipital lobes, and increased Pi/ATP ratio, cerebral Mg, and Pi absolute values were found in the same areas during FAM compared to the control focused attention awake state.

**Conclusions:** Changes in the temporal areas and basal ganglia may be interpreted as a higher energetic state induced by meditation, whereas the frontal and occipital areas showed changes that may be related to a down-regulation in ATP turnover, energy state, and oxidative capacity.

## 1. Introduction

The influence of meditation on brain function is increasingly studied, and possible therapeutic effects are discussed. For example, meditation-based training was proposed as a part of preventing dementia (Chételat et al., 2018). Similarly, short-term intensive meditation was found to have positive effects on resilience, which is known to have prophylactic capabilities on psychiatric disorders (Kwak et al., 2019). Positive effects of this kind could be paving the path for future therapeutic strategies, for example, in depression and anxiety disorders (Hwang et al., 2017).

As one of the basic meditation techniques, focused attention meditation (FAM) is usually one of the first exercises practiced by meditation beginners. Together with open monitoring meditation (OMM), it is considered as a mindfulness-based intervention. FAM is a meditative technique where the meditators focus their attention on a specific target (e.g., breathing), trying to avoid external and internal distractions (Lutz et al., 2008; Yoshida et al., 2020).

Previous studies revealing brain mechanisms associated with meditation techniques dealt mainly with functional MRI (fMRI) (Boccia et al., 2015; Miyoshi et al., 2020), resting-state fMRI (rsfMRI) (Parkinson et al., 2019), electroencephalogram (EEG) (Panda et al., 2016), proton magnetic resonance spectroscopy (^1^H-MRS), and diffusion tensor imaging (DTI) (Fayed et al., 2013).

For example, a study by Fayed et al. (2013) found that the common ^1^H-MRS metabolites, namely, myoinositol (ml), N-acetyl-aspartate (NAA), and glutamate (Glu), are altered in long-term zen meditators. Myoinositol was increased in the posterior cingulate gyrus, while Glu and NAA were lower in the left thalamus. The same study used, besides ^1^H-MRS, DTI on the same group of meditators. DTI showed a lower mean diffusivity in the left parietal white matter (PWM), and the changes correlated with the years of meditation training, indicating not just functional but also the structural changes in the brain of the long-term meditators. In another study with DTI in meditators, Luders et al. (2011) showed changes in the characteristics of white matter (WM) fiber indicating the contribution of meditation to enhanced structural connectivity.

Boccia et al. (2015) analyzed 37 individual fMRI studies and 63 fMRI experimental studies in experienced meditators with a total of 2,294 participants, and summed it up in a meta-analysis. They found evidence that meditation leads to structural and functional brain modifications and activation of brain regions in charge of self-relevant information processing, focused problem-solving, adaptive behavior, self-regulation, interoception, and self-awareness.

In a more recent fMRI study, Miyoshi et al. (2020) examined the fMRI differences between resting and meditative states (induced by FAM) on brain functions in novice meditators. Using a conventional statistical approach, *t*-test results did not show any significant difference in any of the brain regions between the resting state and the meditation state. However, differences appeared in eight brain regions using Tucker3 clustering. These regions included the left inferior frontal gyrus, operculum, the right inferior occipital lobe, the right parahippocampus, the right cerebellum, the right cingulate gyrus, the mid-part, the left cerebellum crus, the left inferior occipital lobe, and the right paracentral lobule.

Another study by Parkinson et al. (2019) examined the rsfMRI functional connectivity related to cognition and attention. Their analysis described increased functional connectivity with regions like anterior cingulate cortex, related to attention control, interoception, and executive function during trait mindfulness. On the contrary, a reduced degree of connectivity was observed for regions related to self-referential processing and mind wandering such as dorsomedial prefrontal cortex in the central executive network component (superior frontal gyrus).

An even earlier study by Panda et al. (2016) used EEG and rsfMRI to compare the spatial extents and temporal dynamics of the default mode network (DMN) during rest and meditation. Their results depicted a decrease in posterior cingulate connectivity and an increase in middle frontal and temporal connectivity in experienced meditators during both rest and, meditation compared to the healthy controls.

From these studies, it can be seen that the most commonly activated areas of the brain during meditation were basal ganglia and frontal, parietal, and temporal areas of the brain. It is not completely understood why this is the case. Basal ganglia are proven to be extremely important in the control of motor functions, motor learning, behavior control, and emotions (Lanciego et al., 2012). These last two functions could especially be important during the meditative practice. The frontal lobe is important in many different roles. Hu et al. (2016) showed the importance of the right superior frontal gyrus in inhibitory control of impulses. Hoffmann (2013) reminded on a role of the frontal lobe in control of working memory, especially in focused attention control.

However, the current literature is lacking studies involving both ^31^P-MRS and meditation. We hypothesized that, using this technique, we will be able to gain insights in to brain energy metabolism during meditation. This technique could give us an insight in exact changes in the relative concentration of important energy metabolites and their ratios during the meditative state. Furthermore, possible changes in pH and changes in magnesium concentration could be observed, both of which are important indicators of energy homeostasis.

Phosphorous magnetic resonance is an MRI technique used to investigate different phosphorus-based energy and membrane metabolites. Some of the phosphorus metabolites that can be detected by ^31^P-MRS are phosphocreatine (PCr), inorganic phosphate (Pi), and (ATP) that represent important indicators of energy metabolism. Furthermore, products of cell membrane metabolism can be detected and are represented by phospholipids: phosphomonoesters (PME) and phosphodiesters (PDE). PME and PDE represent indications of the synthesis and degradation of the cell membrane, respectively. Finally, intracellular pH (Petroff and Prichard, 1983) and the concentration of magnesium (Mg^2+^) (Iotti et al., 1996, 2000) can be calculated. Often, ratios of the various metabolites are reported in order to get results comparable to other studies (Steiger et al., 2018).

This study specifically aimed to compare relative concentrations of each metabolite in ^31^P-MRS, but also their ratios in volunteers under FAM compared to the formal focused attention awake state. This was possible because our participants did not change position between the two measurements that allowed us to have both states of mind in the scanner immediately one after another so the coil loadings for both states were the same. This is important because other, studies where only determination of ratio was possible could only speculate why the ratios were significantly different. Without determining the relative concentration of absolute values and their change, it is impossible to say which constituent of a certain ratio is to "blame" for the change of that ratio. Gizewski et al. (2021) analyzed a longitudinal part of the study with the same participants and could reveal significant differences in brain metabolism between meditation-naive participants and following 7 weeks of meditation exercise. However, that study, like others before, could analyze only ratios and not the absolute values of metabolites due to significant influences of different coil loadings after position changes.

The main aim of this study was therefore to investigate which brain areas show changes in energy metabolism during meditation. We hypothesized that the changes in the brain energy metabolism during meditation as seen on ^31^P-MRS will correspond to the activation patterns seen in other imaging modalities (fMRI and PET). Further, we investigated how relative concentrations of ^31^P-MRS absolute values differ between the focused attention awake state and the meditation state. The technical novelty of this study is, in particular, the possibility to investigate the relative concentration of absolute values of ^31^P metabolites, and not only their ratios.

## 2. Materials and Methods

### 2.1. Volunteers, Inclusion, and Exclusion Criteria

A local yoga school (Yogamood, Innsbruck, Austria) recruited 18 healthy females and 12 males *via* word-of-mouth advertising. Patients under the age of 18, with any medical condition, or with evidence of structural brain abnormality on MRI were excluded. Additionally, usual MRI exclusion criteria were applied (anxiety, claustrophobia, and ferromagnetic implants). All patients were requested to perform 7 weeks of meditation on a daily basis during the study. They were asked to avoid alcohol, caffeine, and nicotine 4 h prior to the MRI scan. The study was conducted in accordance with the Declaration of Helsinki, and the study protocol was approved by the Ethics Committee at the Medical University of Innsbruck. All participants gave written informed consent.

### 2.2. Study Design

This MRI study was designed as an investigation on healthy participants that were initially inexperienced in meditation. Each volunteer was scanned after 7 weeks of the meditation training process, with two consecutive MRI scans. The first time was during meditation (FAM state), and the second time was during focused attention awake state immediately after the first scan, without leaving the scanner. The “focused awake” state was chosen as a control state to be as similar as possible but also being clearly distinct to the meditative part. Therefore, focused attention on a cognitive task, backwards counting, was used as a control state (focused attention awake state). Participants were instructed to keep their eyes closed during both “tasks” (FAM vs. focused attention awake state)—exactly as they have been doing during the 7 weeks of training.

During the meditation training period, an application was used to support and monitor the learning and training processes. After 7 weeks of meditation training, ^31^P-MRS was performed during focused attention meditation state using the learned meditative technique, which means that each participant was in a relaxed state, with closed eyes and focusing on breathing, and then repeated in focused attention awake state (psychic wakefulness due to basic mental arithmetic—counting). The 7 weeks of meditative training was performed as described in the study of Gizewski et al. (2021). All volunteers were scanned during evening hours to account for changes throughout the day.

### 2.3. Meditative Technique and Training Protocol

Under the guidance of an experienced meditation and pranayama teacher, the participants attended a total of 14 sessions of the Raja Yoga technique for 7 weeks. In addition, throughout the study period, participants also performed daily meditation exercises for at least 15 min each day.

In preparation for the MRI investigation, participants practiced 5 min of nadi shodhana visama vritti in sitting position. This alternating breathing technique includes the us age of the thumb and fourth finger to close alternating the right and the left nostril. Visama vritti means that the duration of exhalation was double as long as the inhalation. In the MRI scanner, the participants then practiced the alternating breathing in a lying position without closing the nostrils for 3 min, followed by silent breath observation and yoga nidra (deep relaxation) in this lying position. Yoga nidra is a deep relaxation in lying position, known as “yogic sleep”. This meditation technique was combined with the focused meditation technique, antar mouna including observation of the mind and calming the thoughts of an individual to achieve inner silence (Gizewski et al., 2021).

Focused attention awake state was achieved by steady counting backward.

### 2.4. ^31^P-MRS Acquisition

Phosphorous magnetic resonance spectroscopy was performed on a 3T multi-nuclear whole body system (Skyra, Siemens Medical AG, Erlangen, Germany) with a double-tuned ^1^H/^31^P volume head coil (Rapid Biomedical, Würzburg, Germany). For each participant, a three-dimensional (3D) MRS block of the whole brain was acquired. It was planned on a T2-SPACE sequence [sagittal-oriented, T2-weighted 3D sequence with isotropic resolution and a voxel size of 1.2 x 1.2 x 1.2 mm^3^ (TR = 3,000 ms, TE = 412.0 ms, TA = 2:50)] volume of interest was defined as a field of view of 240 x 240 x 200 mm^3^ and a voxel size of 15 x 15 x 25 mm^3^. The study of ^31^P-MRS was performed with WALTZ 4 proton decoupling, TR 2,000 ms, TE 2.3 ms, flip angle of 60°, and 10 averaged acquisitions similar to that shown in the study by Hattingen et al. (2009).

The ^31^P-MRS sequence (Hattingen et al., 2013; Steiger et al., 2018), employed, implies large voxel sizes and the “voxel bleeding” effect due to a poor point spread function (PSF), as well as contamination with neighboring tissues, cerebrospinal fluid, and bones. However, in order to avoid these contaminations, our MRS sequence was acquired with Hamming weighting, where one can assume that the majority of our signal originates from the effective voxel size, which corresponds with our desired tissue (Korzowski et al., 2020). Additionally, we tried to overcome possible additional contamination of undesired tissue by invariably choosing voxels with more than two-third of the desired tissue involvement. Regarding the proof of a reliable test-retest reproducibility with state-of-the-art MRS methodology at different field strengths, we want to refer to the following research groups (Okada et al., 2007; Terpstra et al., 2016), as it was not possible within the scope of this study to perform more than two MRI scans.

### 2.5. Data Processing and Statistical Analysis

jMRUI software package (version 5.0, http://www.mrui.uab.es) utilizing the non-linear least square fitting algorithm AMARES, which considers prior knowledge (Vanhamme et al., 1997), was used to process all ^31^P-MRS data offline. Twelve Lorentzian-shaped exponentially decaying sinusoids were incorporated for the fitting procedure as follows: phosphocholine, phosphoethanolamine (the sum of both referred to as PME), (Pi), glycerophosphocholine, glycerophosphoethanolamine (the sum of both referred to as PDE), (PCr), and (ATP) consisting of two doublets (γ-ATP and α-ATP) and triplets (β-ATP), which were added up. The peak of β-ATP can be used as an internal quantification, as it can be considered uncontaminated by α-ATP, nicotinamide adenine dinucleotide (NAD) and NAD hydrogen (NADH) (oxidized) contributions to α-ATP (Du et al., 2007). However, we calculated the mean of α-ATP, β-ATP, and γ-ATP as our reference values and termed it ATP. All spectra were visually checked for artifacts and their quality according to the criteria of existing literature (Kreis, 2004) by a physicist. Voxels that did not fulfill quality criteria were excluded from further analysis.

We calculated the parameters of Mg^2+^ and pH according to the formulas from Iotti et al. (1996, 2000) as follows: The changes in Mg^2+^ concentrations were estimated from the chemical shift difference between the β-ATP and the PCr signal (Δβ).

(1) $$\begin{array}{l} \;pMg=4.24-log_{10}\left[\frac{{\left(18.58+\Delta \beta \right)}^{0.42}}{{\left(-15.74-\Delta \beta \right)}^{0.84}}\right] \end{array}$$

The pH value was determined from the signal position of (ΔPi) regarding to PCr, set as the main reference. pH was the calculated according the formula by Petroff and Prichard (1983):

(2) $$\begin{array}{l} \;pH=6.706-0.0307\left[Mg\right]+log_{10}\left[\frac{\left(\Delta Pi-3.245\right)}{\left(5.778-\Delta Pi\right)}\right] \end{array}$$

Five designated brain areas (frontal, parietal, occipital, temporal, and basal ganglia) were analyzed with one or more ^31^P-MRS voxels in each volunteer (see Supplementary Material, similar as the study of Gizewski et al., 2021). The amount of the included voxels per area depended on the size of the respective area and the quality of the spectra. Each single spectrum was visually evaluated according to the criteria postulated by Kreis (2004), and metabolite ratios were calculated subsequently. The ratios, and the absolute values of PCr, ATP, and Pi, were calculated are as follows: PCr/ATP, PCr/Pi, Pi/ATP.

Statistical analysis of ratios and absolute values was performed using GraphPad Prism (GraphPad Inc., San Diego, CA, USA) and R (R Core Team v. 3.6.1). Data normality of metabolite ratios was assessed with the one-sample Kolmogorov-Smirnov test at a 5% significance level. Outliers were identified and removed with the ROUT method. As the data for metabolite ratios were not normally distributed, a nonparametric Mann-Whitney U test was therefore applied for the investigation of statistically significant differences between the groups. On the contrary, all data were normally distributed for absolute values. Therefore, an unpaired *t*-test was applied to investigate the differences between the groups. Benjamini-Hochberg procedure was performed to control the false discovery rate (FDR).

## 3. Results

### 3.1. Participants

For this study, a total of 30 healthy subjects were enrolled (n = 12 male; n = 18 female); however, as two male and one female had to be excluded due to non-fulfillment of the inclusion criteria, full data sets were finally available for n = 27 subjects. The mean age was 43.3 years (range: 22–69 years).

### 3.2. ^31^P-MRS Results

The results of ^31^P-MRS are shown in Table 1.

**Table 1:** Phosphorous magnetic resonance spectroscopy (^31^PMRS) results: *P*-values after correction were given for areas with significant difference between focused attention meditation (FAM) and focused attention awake state.

**p values (focused attention meditation vs. focused attention awake state)**								
**Brain regions**	Pi	PCr	ATP	pH	Mg	PCr/ATP	Pi/ATP	PCr/Pi
Entire brain	NS	NS	NS	**0.000124**	*0.002048*	**0.037714**	NS	**0.002048**
Right brain	NS	NS	NS	**0.032772**	*0.000124*	**0.038981**	NS	NS
Left brain	*0.002784*	NS	NS	**0.000124**	*0.032305*	**0.038981**	NS	NS
Basal ganglia	NS	NS	NS	**0.006600**	*0.000124*	**0.001100**	NS	NS
Right basal ganglia	NS	NS	NS	NS	*0.011742*	**0.022129**	NS	NS
Left basal ganglia	NS	NS	NS	**0.036630**	*0.046851*	**0.046851**	NS	NS
Frontal brain	*0.008027*	NS	NS	**0.004243**	*0.000124*	NS	*0.031350*	**0.013559**
Right frontal lobe	*0.036630*	NS	NS	NS	NS	NS	NS	NS
Left frontal lobe	*0.010607*	NS	NS	**0.046487**	*0.000124*	NS	*0.001100*	**0.000124**
Occipital brain	NS	NS	NS	NS	NS	**0.003600**	NS	**0.019008**
Right occipital lobe	NS	NS	NS	NS	NS	**0.019008**	NS	**0.002784**
Left occipital lobe	NS	NS	NS	**0.031821**	*0.043200*	NS	NS	NS
Parietal brain	NS	NS	NS	**0.037714**	NS	NS	NS	NS
Right parietal lobe	NS	NS	NS	NS	NS	NS	NS	NS
Left parietal lobe	NS	NS	NS	**0.000124**	**0.002784**	NS	NS	NS
Temporal brain	NS	NS	NS	NS	*0.023417*	NS	NS	NS
Right temporal lobe	NS	NS	NS	NS	*0.004243*	NS	NS	NS
Left temporal lobe	NS	NS	NS	NS	NS	NS	NS	NS

*Bolded p-values: mean/median is lower in meditation state. Italic p-values: mean/median is higher in meditation state. NS, not significant*.

### 3.3. Absolute Values

Significantly higher Pi values during focused attention meditation state were found in the left hemisphere, the frontal brain, the right frontal lobe, and the left frontal lobe. Significantly higher ATP values during focused attention meditation state were found in the entire brain, the left hemisphere, and the left occipital lobe; however, these results were not significant after correction for FDR. No significantly different PCr values between focused attention meditation and focused attention awake states were found in any region.

### 3.4. Ratios

Significantly lower PCr/ATP ratio during FAM was found in the entire brain, the right hemisphere, the left hemisphere, basal ganglia, right and left basal ganglia individually, occipital brain, and the right occipital lobe.

Significantly higher Pi/ATP ratio during FAM state was found in the frontal brain and the left frontal lobe.

PCr/Pi ratio was significantly lower during FAM in the entire brain, the frontal brain, the left frontal lobe, the occipital brain, and the right occipital lobe.

### 3.5. Intracellular pH and Cerebral Magnesium Levels

During FAM state, intracellular pH was significantly lower in the entire brain, left and right hemispheres, in whole basal ganglia and the left basal ganglia, frontal brain and left frontal lobe, in parietal brain, and left parietal lobe.

Significantly higher levels of cerebral magnesium during FAM state were found in the entire brain, the right hemisphere, the left hemisphere, basal ganglia, left and right basal ganglia individually, the frontal brain, the left frontal lobe, the left occipital lobe, the temporal brain, and the right temporal lobe. Left parietal lobe is the only brain region where cerebral Mg levels were significantly lower during the meditative state. This is also the only region and only metabolite that was "out of trend" in this research study.

False positive rate calculated with Benjamini-Hochberg procedure was 30.4 %. For detailed results including mean (SD) or median [interquartile range (IQR)] values. see Supplementary Material.

## 4. Discussion

The results of this study show the influence of short-term learned meditation on cerebral energy metabolism. Various changes in important cerebral metabolites during meditation are revealed, such as changes in intracellular pH, changes in ratios representing PCr buffering and ATP turnover, and changes in relative concentration of important absolute values of metabolite like Pi that could represent changes in energy metabolism.

Response to brain activation was always a hot topic in the research world. Simple PubMed search shows how interest in brain activation patterns rises during time. However, brain activation in ^31^P-MRS is difficult to observe. Research study by van de Bank et al. (2018) showed no change in ^31^P-MRS metabolites during visual stimulation in 7T. This emphasizes the difficulties in ^31^P-MRS, especially in "lower" magnetic field strengths like 3T. Nevertheless, detected changes in ^31^P MRS metabolites and ratios can be used for generation of further hypothesis to be tested in larger cohorts and higher field strength.

### 4.1. Absolute Values and Ratios of Metabolites

Regarding the PCr/ATP ratio, we found that the entire brain had significantly lower ratios during the FAM state. PCr and ATP were not significantly different in any of the samples. Right brain had a significantly lower PCr/ATP ratio. Left hemisphere PCr/ATP was lower during the FAM. This region had higher ATP absolute values during FAM state before correction for FDR. Basal ganglia had significantly lower PCr/ATP ratios during FAM state even though no statistically significant difference was found in absolute values in this brain area before or after FDR. The occipital brain had significantly lower PCr/ATP, same as the right occipital lobe. However, left occipital lobe was the only measurement in the occipital cluster that had significantly higher absolute value of ATP before correction for FDR. The main biological role of the PCr is the buffer control of the ATP and support for the demand for energy. ATP, on the other hand, is regarded as the energy currency of the cell (Guimarães-Ferreira, 2014). Lower PCr/ATP ratio in targeted brain regions could be explained with higher energy demand during meditation in these areas, possibly because of increased spending of PCr buffer supplies. Boccia et al. (2015) showed increased activity measured using fMRI in areas such as the thalamus, the caudate nucleus, the lentiform nucleus, or the middle occipital gyrus. Kozasa et al. (2012) in their fMRI study showed that the non regular meditators had higher activity in, among others, lentiform nucleus in comparison with the regular meditators. These results suggest the important role of basal ganglia in the meditation process. Panda et al. (2016) showed an increase in frontal and temporal areas in experienced meditators during meditation using fMRI and EEG.

Inorganic phosphate is also a involved in the metabolism of cell energy (Verma et al., 2016). In general, we found significantly higher ratios of Pi/ATP in the frontal brain and left frontal lobe. These brain areas also had significantly higher Pi absolute values during the focused attention meditation state.

PCr/Pi ratios were significantly lower during the FAM state in the entire brain, the frontal brain, and the left frontal lobe, occipital brain and the right occipital lobe.

All of these areas correspond to the areas found to be active during meditation by the meta-analysis of the fMRI studies, which was carried out by Boccia et al. (2015). They found that areas especially active during meditation are the precuneus, the anterior cingulate cortex, the insula, the premotor cortex, and the superior frontal gyrus.

It could be assumed that the ratios of the ^31^P metabolites in the occipital, parietal, and frontal lobes are lower during FAM because of the higher activity of these areas. Significantly higher value of Pi during FAM in frontal areas also matches this assumption.

### 4.2. Cerebral Magnesium and pH

One general finding in the investigated brain areas was that intracellular pH was generally significantly lower during the FAM state as compared to the focused attention awake control state. This difference was found in the entire brain, the left and right hemispheres, whole basal ganglia left basal ganglia, the frontal brain, and the left frontal lobe, the left occipital lobe, the parietal brain, and the left parietal lobe. It is still not clear how exactly the pH of the brain corresponds to its function (Magnotta et al., 2012). Both intracellular and extracellular pH rapidly changes during brain activation (Chesler, 2003). Exact mechanisms are still debated. As for intracellular pH, its disturbance was demonstrated in some pathological conditions like stroke (Orlowski et al., 2011) or acute neuroinflammation in mice (Tyrtyshnaia et al., 2016). Intracellular pH in relation to physiological activation was not extensivelly studied. Khlebnikov et al. (2017) report on difficulties surrounding the detection of changes in pH during visual stimuli with existing technologies. van de Bank et al. (2018) also found no change in pH in ^31^P-MRS during visual stimulation. Hendriks et al. (2019) proposed a new method for measuring pH in mitochondrial or extracellular compartments during visual stimulus. Using this method, they showed a minimal shift of the downfield Pi peak toward the main Pi peak during visual stimuli, but these changes remained small and the authors recommended further validation. On the contrary, Dienel and Hertz (2001) report a probable increase in lactate concentration during brain activation, which was shown to correlate with a decrease in pH Paschen et al. (1987). Hagihara et al. (2021) showed a strong negative correlation between brain pH and lactate levels in neuropsychiatric animal models.

The results regarding intracellular pH partly correlate with the results in other activated regions. These results give us an insight into how the intracellular pH could react during the physiological activation of various brain regions.

Iotti et al. (2000) and Iotti and Malucelli (2011) provide evidence that cerebral Mg is involved in the metabolism of cerebral energy, and its levels are tightly regulated by the brain cells. Cerebral Mg levels were almost always higher during FAM, except in the left parietal lobe where they were lower. Higher Mg levels are therefore seen in the entire brain, right and left hemispheres individually, whole basal ganglia with right and left basal ganglia individually, frontal brain and right and left frontal lobes individually, left occipital lobe, temporal brain, and right temporal lobe. Exact correlation of cerebral Mg and brain activation is still a matter of dispute, but several points are already known. Iotti et al. (1996) showed that *in vivo* ATP is mainly present in form of MgATP. From this conclusion, it is clear why magnesium is crucial in ATP homeostasis.

To summarize, our results show changed metabolite measures, namely PCr/ATP ratio, Pi/ATP ratio, PCr/Pi ratio, absolute values of Pi and ATP, and intracellular pH and Mg values, mainly in frontal, parietal, and occipital lobes, and in basal ganglia. These areas are also found to be active on fMRI during meditation process by several studies, as concluded in the meta-analysis by Boccia et al. (2015).

Magan et al. (2019) in the FDG-PET 18-fluorodeoxyglucose-(FDG-PET) study of 12 long term meditators showed the activation of the fronto–parieto–temporal regions. Even though this study is based on short-term meditators, it is interesting to see how quickly the energy metabolism changes. Along these lines, the impact of even a short-term meditation on various brain qualities (e.g., gray matter density or resting-state activity) was shown in a study by Dodich et al. (2018). They concluded that even a short-term meditation training had a significant impact on the brain regions associated with attention, self-control, and self-awareness.

Another large meta-analysis on fMRI and PET findings during meditation, conducted by Fox et al. (2016), showed several interesting points for focused attention meditation. The areas that were activated during focused attention meditation were areas of the brain associated with cognitive control and self-reflection. Activation was found in the premotor cortex, dorsal anterior cingulate cortex, and dorsolateral prefrontal cortex, similar to our findings in frontal and parietal brain areas.

We can conclude that the ^31^P-MRS showed altered phosphorous metabolites in the frontal, occipital, and partly parietal cortex, and basal ganglia. These results extend the results of other neuroimaging studies, by providing evidence for altered energy metabolism in brain during FAM, in areas that correspond to the findings of previous studies. Furthermore, this study gives insights in to changes of relative concentrations of energy metabolites and their ratios during FAM in previously inexperienced short-term meditators.

### 4.3. Limitations of the Study

Activation studies with ^31^P-MRS are relatively unreliable and not sensitive. Even studies at higher magnetic field strengths like 7 T failed to find any difference during brain activation (van de Bank et al., 2018). Technical difficulties in ^31^P-MRS imaging are well known (Liu et al., 2017).

Further, our subjects had only short-term meditation training. This could reduce the effect of meditation on a human brain and diminish our MR signal. Certainly, difficulty in guaranteeing task compliance during scanning is another possible limitation.

Also, we had the assumption that the meditative state would result in a prolonged change in cerebral processing, which did not allow to control order effects.

For future research, we propose a study with a higher number of subjects, experienced in both long- and short-term meditation.

## 5. Conclusions

This study used ^31^P-MRS to assess relative concentrations of brain energy metabolites, and not only their ratios, in meditators during FAM in comparison with focused attention on counting (focused attention awake state).

The results of the current study indicate that even short-term training in focused attention meditation has effects on brain energy state. Changes in the basal ganglia and temporal lobes may be viewed as a higher energetic state induced by meditation, and the occipital and frontal lobes showed changes that may be related to a down-regulation in ATP turnover, energy state and oxidative capacity. In fact, additional data may have to be generated and further studies conducted to explain the role specific regions have during meditation.

## Data Availability Statement

The raw data supporting the conclusions of this article will be made available by the authors, without undue reservation.

## Ethics Statement

The studies involving human participants were reviewed and approved by Ethical Committee Medical University of Innsbruck. The patients/participants provided their written informed consent to participate in this study.

## Author Contributions

MG, RS, MW, AG, NS, and EG planned the study and wrote the study protocol. MW and NS led the meditation training. RS, MG, and PS acquired the MRS data. MG and RS analyzed the data. MG and MR conducted the statistical analysis. MG, RS, AG, and EG analyzed the results and put it in context of current scientific understanding. MG and RS wrote the manuscript. All authors read the manuscript and agreed on its publication.

## Conflict of Interest

MG was employed by the company VASCAge GmbH. The remaining authors declare that the research was conducted in the absence of any commercial or financial relationships that could be construed as a potential conflict of interest.
