# Supplementary material for: Brain Energy Metabolism in Two States of Mind Measured by Phosphorous Magnetic Resonance Spectroscopy
Source: Front Hum Neurosci. 2021 Jun 28;15:686433. doi: 10.3389/fnhum.2021.686433 (PMC8273761; doi:10.3389/fnhum.2021.686433)
Supplement: Supplementary file 1 [file Data_Sheet_1.PDF]

Supplementary Material

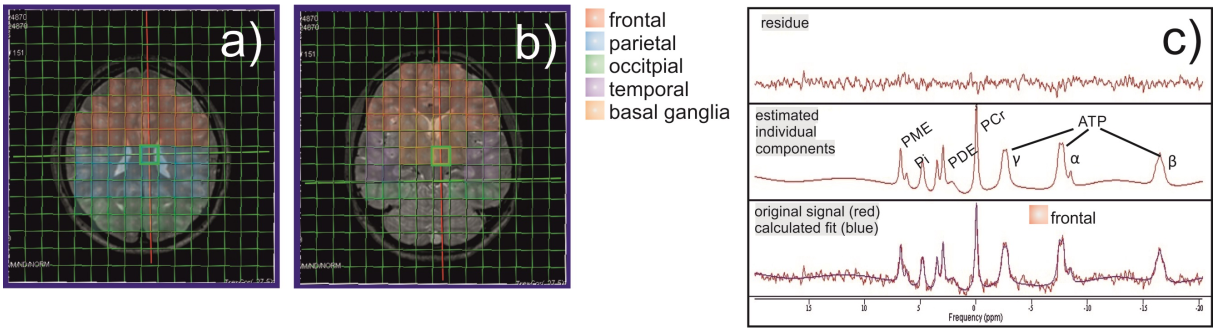

Figure S1. For each participant five different brain regions where chosen to be investigated (a, b in color marked), and exemplary one spectrum taken from one voxel in the frontal region (c).

Absolute values- focused attention meditation vs focused attention awake state)									
	**pI (a.u.)**			**PCr (a.u.)**			**ATP (a.u.)**		
	**p value**	**mean (SD)**		p value	**mean (SD)**		p value	**mean (SD)**	
**Brain regions**		**Meditation**	**Awake**		**Meditation**	**Awake**		**Meditation**	**Awake**
Entire brain	NS	x	x	NS	x	x	NS	x	x
Right brain	NS	x	x	NS	x	x	NS	x	x
Left brain	*0.002784*	134.1 (31.25)	127.5 (33.67)	NS	x	x	NS	x	x
Basal ganglia	NS	x	x	NS	x	x	NS	x	x
Right basal ganglia	NS	x	x	NS	x	x	NS	x	x
Left basal ganglia	NS	x	x	NS	x	x	NS	x	x
Frontal brain	*0.008027*	122.1 (31.42)	117.2 (31.81)	NS	x	x	NS	x	x
Right frontal lobe	*0.036630*	125.7 (26.98)	117.9 (27.49)	NS	x	x	NS	x	x
Left frontal lobe	*0.010607*	123.5 (31.89)	116.7 (32.47)	NS	x	x	NS	x	x
Occipital brain	NS	x	x	NS	x	x	NS	x	x
Right ocicpital lobe	NS	x	x	NS	x	x	NS	x	x
Left ocicpital lobe	NS	x	x	NS	x	x	NS	x	x
Parietal brain	NS	x	x	NS	x	x	NS	x	x
Right parietal lobe	NS	x	x	NS	x	x	NS	x	x
Left parietal lobe	NS	x	x	NS	x	x	NS	x	x
Temporal brain	NS	x	x	NS	x	x	NS	x	x
Right temporal lobe	NS	x	x	NS	x	x	NS	x	x
Left temporal lobe	NS	x	x	NS	x	x	NS	x	x

Table S1. Mean values and standard deviations for data with statistical significance between focused attention meditation and focused attention awake state. **Bolded** p-values: mean/median is lower in meditation state. *Italic* p-values: mean/median is higher in meditation state. NS: not significant. a.u. - arbitrary units

Ratios and calculated values - focused attention meditation vs focused attention awake state)															
	**pH**			**Mg (a.u.)**			**PCr/ATP**			**Pi/ATP**			**PCr/Pi**		
	**p value**	**median (IQR)**		p value	**median (IQR)**		p value	**median (IQR)**		p value	**median (IQR)**		p value	**median (IQR)**	
**Brain regions**		**Meditation**	**Awake**		**Meditation**	**Awake**		**Meditation**	**Awake**		**Meditation**	**Awake**		**Meditation**	**Awake**
Entire brain	**0.000124**	7.045 (0.027)	7.048 (0.029)	*0.002048*	0.1021 (0.0107)	0.1015 (0.0106)	**0.037714**	1.14 (0.271)	1.16 (0.286)	NS	x	x	**0.002048**	3.564 (1.009)	3.628 (1.035)
Right brain	**0.032772**	7.046 (0.027)	7.047 (0.029)	*0.000124*	0.1031 (0.0117)	0.1015 (0.01225)	**0.038981**	1.145 (0.278)	1.172 (0.29)	NS	x	x	NS	x	x
Left brain	**0.000124**	7.045 (0.02)	7.05 (0.029)	*0.032305*	0.102 (0.0109)	0.1016 (0.0102)	**0.038981**	1.284 (0.229)	1.312 (0.21)	NS	x	x	NS	x	x
Basal ganglia	**0.006600**	7.041 (0.026)	7.046 (0.024)	*0.000124*	0.1005 (0.0088)	0.0984 (0.0094)	**0.001100**	1.195 (0.192)	1.238 (0.152)	NS	x	x	NS	x	x
Right basal ganglia	NS	x	x	*0.011742*	0.101 (0.0094)	0.09815 (0.0104)	**0.022129**	1.183 (0.186)	1.232 (0.138)	NS	x	x	NS	x	x
Left basal ganglia	**0.036630**	7.041 (0.028)	7.048 (0.024)	*0.046851*	0.1 (0.00885)	0.0985 (0.009)	**0.046851**	1.221 (0.196)	1.243 (0.152)	NS	x	x	NS	x	x
Frontal brain	**0.004243**	7.04 (0.027)	7.043 (0.03)	*0.000124*	0.1012 (0.0102)	0.0998 (0.01017)	NS	x	x	*0.031350*	0.33 (0.099)	0.323 (0.086)	**0.013559**	3.378 (0.946)	3.478 (0.885)
Right frontal lobe	NS	x	x	NS	x	x	NS	x	x	NS	x	x	NS	x	x
Left frontal lobe	**0.046487**	7.039 (0.027)	7.042 (0.03)	*0.000124*	0.1022 (0.0111)	0.0996 (0.01052)	NS	x	x	*0.001100*	0.3375 (0.1032)	0.323 (0.0815)	**0.000124**	3.35 (0.842)	3.753 (0.865)
Occipital brain	NS	x	x	NS	x	x	**0.003600**	1.27 (0.195)	1.303 (0.17)	NS	x	x	**0.019008**	3.94 (1.032)	4.068 (1.221)
Right occipital lobe	NS	x	x	NS	x	x	**0.019008**	1.276 (0.206)	1.325 (0.193)	NS	x	x	**0.002784**	3.926 (1.129)	4.24 (1.305)
Left occipital lobe	**0.031821**	7.053 (0.0023)	7.06 (0.029)	*0.043200*	0.1037 (0.01197)	0.1007 (0.0097)	NS	x	x	NS	x	x	NS	x	x
Parietal brain	**0.037714**	7.044 (0.023)	7.047 (0.0269)	NS	x	x	NS	x	x	NS	x	x	NS	x	x
Right parietal lobe	NS	x	x	NS	x	x	NS	x	x	NS	x	x	NS	x	x
Left parietal lobe	**0.000124**	7.042 (0.024)	7.048 (0.022)	**0.002784**	*0.0995* (0.0082)	0.102 (0.00795)	NS	x	x	NS	x	x	NS	x	x
Temporal brain	NS	x	x	*0.023417*	0.1082 (0.018)	0.1057 (0.0136)	NS	x	x	NS	x	x	NS	x	x
Right temporal lobe	NS	x	x	*0.004243*	0.1092 (0.0201)	0.1051 (0.0146)	NS	x	x	NS	x	x	NS	x	x
Left temporal lobe	NS	x	x	NS	x	x	NS	x	x	NS	x	x	NS	x	x

Table S2. Median values and interquartile range for metabolite ratios and calculated data with statistical significance between focused attention meditation and focused attention awake state. **Bolded** p-values: mean/median is lower in meditation state. *Italic* p-values: mean/median is higher in meditation state. NS: not significant. a.u. - arbitrary units
